# Pulmonary Embolism after Arthroscopic Rotator Cuff Repair: A Case Report

**DOI:** 10.1155/2013/801752

**Published:** 2013-02-28

**Authors:** Tadashi Yamamoto, Kazuya Tamai, Miwa Akutsu, Kazuo Tomizawa, Takuya Sukegawa, Yutaka Nohara

**Affiliations:** Department of Orthopaedic Surgery, Dokkyo Medical University, 880 Kitakobayashi, Mibu, Tochigi 321-0293, Japan

## Abstract

Total hip/knee arthroplasty may cause venous thromboembolism (VTE) as a postoperative complication. However, there are few reports on VTE after arthroscopic shoulder surgery. We report a patient who developed pulmonary embolism (PE) 6 days after arthroscopic rotator cuff repair but recovered without sequelae. In this case, the possibility of DVT of the lower limbs was denied by contrast-enhanced CT. Most possibly, the source of PE was deep vein thrombosis (DVT) of the upper limb under Desault fixation which showed arthroscopic surgery-related swelling postoperatively.

## 1. Introduction

Total hip/knee arthroplasty may cause venous thromboembolism (VTE) as a postoperative complication. However, there are few reports on VTE after arthroscopic shoulder surgery; its incidence is reportedly less than 0.01% [1]. We report a patient who developed pulmonary embolism 6 days after arthroscopic rotator cuff repair. The patient gave an informed consent for publication.

## 2. Case Report

A 72-year-old female was referred to us with the right shoulder pain, which had persisted for 2 months after lifting a heavy pot. On the initial consultation, the height, body weight, and body mass index (BMI) were 147 cm, 46 kg, and 21 kg/m^2^, respectively. The ranges of motion of the right shoulder on flexion, external rotation, and internal rotation were 60 degrees, 30 degrees, and L2, respectively. A supraspinatus test showed a positive reaction on the right side.

**Figure 1:**
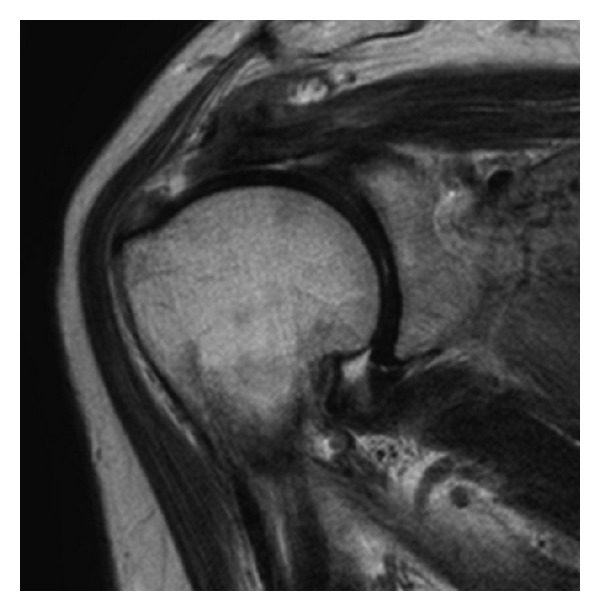
T2-weighted magnetic resonance image of the right shoulder. Tear of the supraspinatus tendon is noted.

The patient had a history of hypertension and arrhythmia. However, there were no electrocardiographic abnormalities on preoperative examination. Magnetic resonance imaging (MRI) revealed rupture of the right supraspinatus tendon (Figure 1). Arthroscopic rotator cuff repair was performed.

**Figure 2:**
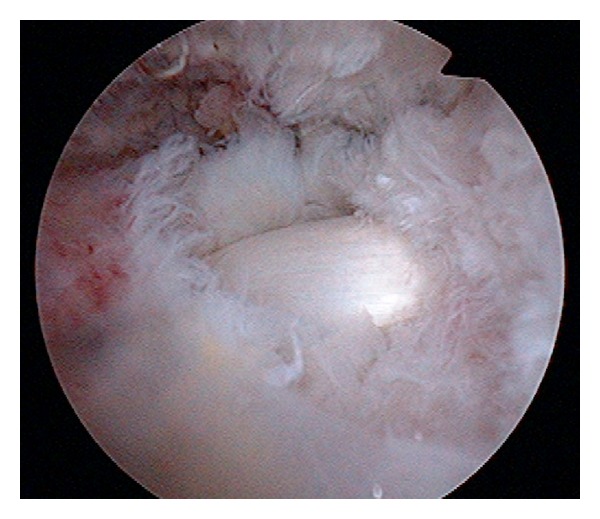
Arthroscopic view of the torn rotator cuff. A medium-sized tear, 15 mm in diameter, of the supraspinatus tendon was seen via a posterolateral portal to the subacromial space. The long tendon of the biceps was noted through the torn portion of the supraspinatus.

Under general anesthesia and brachial plexus block, the patient was put in a 60-degree beach-chair position. During surgery, intermittent air compression was performed to prevent deep vein thrombosis (DVT) of the lower limbs. Perfusion pressure of the shoulder ranged from 40 to 80 mmHg. At the attachment site of the supraspinatus tendon, a 1.5-cm rupture was observed (Figure 2). Using a suture anchor, the supraspinatus tendon was repaired. The duration of surgery was 3 hours and 8 minutes. The duration of anesthesia was 4 hours and 31 minutes. After surgery, Désault fixation was performed for the right upper limb.

**Figure 3:**
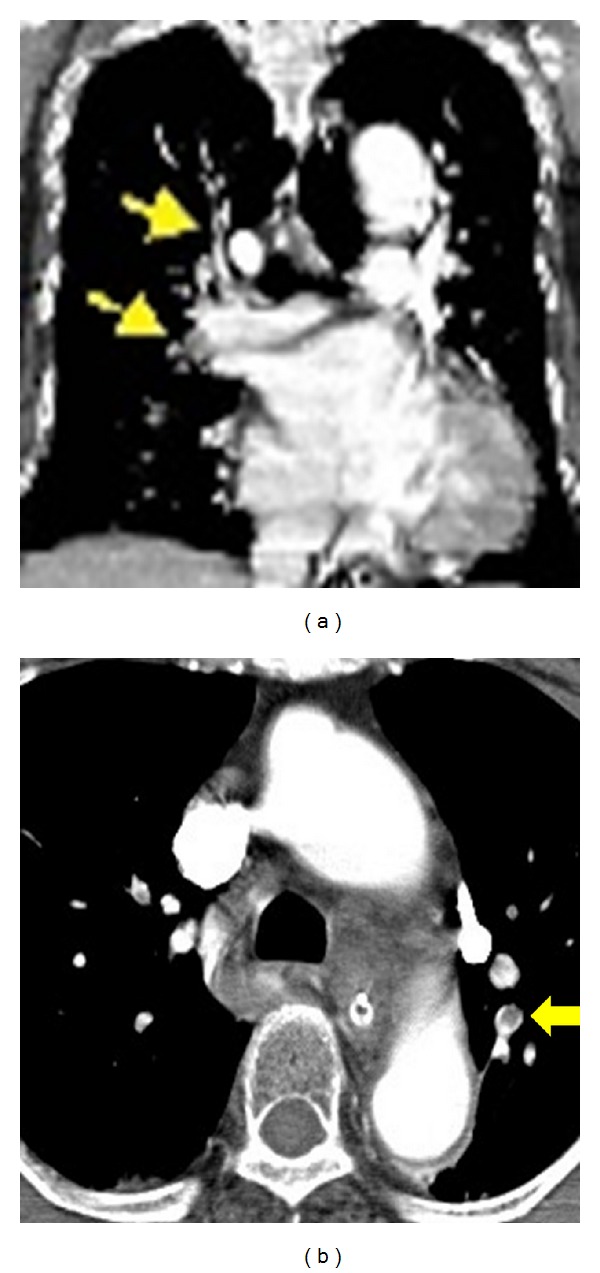
Contrast-enhanced CT of the lung. The coronal reconstructed image (a) revealed thrombi in the superior and inferior lobe branches of the right pulmonary artery (yellow arrows). The transverse image (b) showed a thrombus in the superior lobe branch of the left pulmonary artery (yellow arrow).

The patient ambulated the day after surgery. Pendulum exercise was started 3 days after surgery. Six days after surgery, she was found in a cardiac arrest in the lobby of the ward. Resuscitation was promptly conducted. Blood test showed that the D-dimer level was high, 30 *μ*g/mL. Under a tentative diagnosis of pulmonary embolism (PE), contrast-enhanced computed tomography (CT) was performed. However, neither PE nor DVT was suggested. Electrocardiography showed an increase in the ST level in II, III, and aVf leads. Intracardiac catheterization was conducted. However, there were no abnormal findings; the possibility of acute myocardial infarction was ruled out. The following day, additional contrast-enhanced CT revealed thrombi in the superior and inferior lobe branches of the right pulmonary artery and in the superior lobe branch of the left pulmonary artery (Figure 3). There was no DVT of the lower limbs. However, for routine contrast-enhanced CT in our hospital, the trunk and lower limbs were examined; therefore, contrast-enhanced CT of the upper limbs was not performed.

Promptly, the administration of heparin at 10,000 U/day was started. The D-dimer level decreased to 4.3 *μ*g/mL. Furthermore, the respiratory state improved, and extubation was conducted. The dose of heparin was decreased to 5,000 U/day 6 days after the onset of PE. Warfarin administration at 3 mg/day was initiated. Eight days after the onset of PE, contrast-enhanced CT confirmed the disappearance of the pulmonary arterial thrombi. With monitoring the PT-INR, the dose of warfarin was decreased to 2.75 mg/day. The patient was discharged without sequelae 24 days after the onset of PE.

Detailed examination of thrombotic predispositions, such as antithrombin deficiency, prothrombin disturbance, protein C/S deficiency, factor V dysfunction, and anti-phospholipid antibody syndrome, was performed in the Department of Hematology. However, there were no abnormalities. During the 1-year-and-3-month postoperative followup, there has been no sign of recurrent PE.

## 3. Discussion

Jameson et al. reported that the incidence of DVT related to arthroscopic shoulder surgery was less than 0.01%, and that the incidence of PE was also less than 0.01% [1]. According to the guideline for VTE prevention proposed by the Japanese Orthopaedic Association [2], no patient with VTE after upper limb surgery was reported in Japan between 1990 and 2001. After 2002, 6 patients with VTE after upper limb surgery (2 with DVT, 4 with PE, including 1 with fatal PE) have been reported [2]. However, none of these 6 patients had undergone arthroscopic shoulder surgery.

Reviewing the English literature, we found 9 patients with VTE after arthroscopic shoulder surgery [3–9]. There were 7 males and 2 females, with a mean age of 46 years. The mean interval from surgery until onset was 6.4 days. DVT of the lower limbs was noted in 2 patients, DVT of the upper limbs in 6, and PE in 5 (Table 1) [3–9]. In 8 of the 9 patients, risk factors for DVT, that is, obesity, malignant disorders such as Hodgkin's lymphoma, thrombotic predispositions (antiphospholipid antibody syndrome, prothrombin disturbance, and factor V dysfunction), and preoperative driving for a long duration, were present (Table 1) [3–9].

In our patient, there was no risk factor for DVT before surgery. Furthermore, the source of PE was unclear. The possibility of DVT of the lower limbs was ruled out. However, we cannot rule out the possibility that arthroscopic surgery-related swelling of the upper limbs and postoperative Désault fixation caused DVT of the upper limbs. In addition, the duration of surgery was prolonged, 3 hours and 8 minutes. There are few reports that discussed the relationship between operation time and perioperative PE in orthopedics surgery, while Sakon et al. reported prolonged abdominal surgery as a risk factor of perioperative PE [10]. In the current case, a longer operation time than usual could have enhanced the swelling of the upper limb, thus increasing the risk of DVT.

Positive exercise of the lower limbs, elastic stocking use, and intermittent air compression are known to be useful for preventing DVT of the lower limbs [2]. It remains to be clarified, however, whether or not positive exercise and elastic bandages are useful for preventing DVT of the upper limbs, as demonstrated for the lower limbs. Similarly, anticoagulation therapy using enoxaparin, for example, which is also effective for preventing DVT in the lower limb surgeries [11], needs to be tested for its effectiveness to prevent DVT of the upper limbs. In the clinical practice, we would recommend serial D-dimer measurements in the perioperative period for detecting DVT/PE even in the arthroscopic shoulder surgery.

## 4. Conclusion

Arthroscopic shoulder surgery can cause PE.

## Figures and Tables

**Table 1:** Reported cases of VTE following arthroscopic shoulder surgery.

Author year [Reference no.]	Age (years) Gender	DVT of lower limb	DVT of upper limb	PE	Interval to onset (days)	Risk factor
Burkhart 1990 [3]	32 male	No	Yes	No	3	Hodgkin's lymphoma
Saleem and Markel 2001 [4]	68 male	Yes	No	Yes	1	Eight-hour seated car drive before surgery
Polzhofer et al. 2003 [5]	48 male	No	Yes	Yes	7	Obesity
Creighton and Cole 2007 [6]	43 male	No	Yes	Yes	7	None
Cort$\overset{´}{\text{e}}$s et al. 2007 [7]	52 female	No	No	Yes	10	Obesity; estrogen intake
Hariri et al. 2009 [8]	25 male	No	Yes	Yes	10	None
	30 male	No	Yes	No	4	Antiphospholipid antibody syndrome
Bongiovanni et al. 2009 [9]	54 female	Yes	No	No	10	Prothrombin disturbance
	66 male	No	Yes	Yes	6	Factor V dysfunction
